# Direct Diabetes-Related Costs in Young Patients with Early-Onset, Long-Lasting Type 1 Diabetes

**DOI:** 10.1371/journal.pone.0070567

**Published:** 2013-08-13

**Authors:** Christina Bächle, Andrea Icks, Klaus Straßburger, Marion Flechtner-Mors, Andreas Hungele, Peter Beyer, Kerstin Placzek, Ulrich Hermann, Andrea Schumacher, Markus Freff, Anna Stahl-Pehe, Reinhard W. Holl, Joachim Rosenbauer, on behalf of the DPV Initiative and the German BMBF Competence Network Diabetes Mellitus

**Affiliations:** 1 Institute for Biometrics and Epidemiology, German Diabetes Center, Leibniz Institute for Diabetes Research at Heinrich Heine University, Düsseldorf, Germany; 2 Department of Public Health, Faculty of Medicine, Heinrich-Heine-University, Düsseldorf, Germany; 3 Institute of Epidemiology and Medical Biometry, University of Ulm, Ulm, Germany; 4 Clinic for Children and Adolescents, Evangelisches Krankenhaus, Oberhausen, Germany; 5 Pediatric and Adolescent Medicine, University Hospital, Martin-Luther University, Halle, Germany; 6 Pediatric Diabetes Practice, Reutlingen, Germany; 7 Clinic for Child and Adolescent Medicine, Klinik am Eichert, Göppingen, Germany; 8 Princess Margaret Children's Clinic, Darmstadt, Germany; Groningen Research Institute of Pharmacy, United States of America

## Abstract

**Objective:**

To estimate diabetes-related direct health care costs in pediatric patients with early-onset type 1 diabetes of long duration in Germany.

**Research Design and Methods:**

Data of a population-based cohort of 1,473 subjects with type 1 diabetes onset at 0–4 years of age within the years 1993–1999 were included (mean age 13.9 (SD 2.2) years, mean diabetes duration 10.9 (SD 1.9) years, as of 31.12.2007). Diabetes-related health care services utilized in 2007 were derived from a nationwide prospective documentation system (DPV). Health care utilization was valued in monetary terms based on inpatient and outpatient medical fees and retail prices (perspective of statutory health insurance). Multiple regression models were applied to assess associations between direct diabetes-related health care costs per patient-year and demographic and clinical predictors.

**Results:**

Mean direct diabetes-related health care costs per patient-year were €3,745 (inter-quartile range: 1,943–4,881). Costs for glucose self-monitoring were the main cost category (28.5%), followed by costs for continuous subcutaneous insulin infusion (25.0%), diabetes-related hospitalizations (22.1%) and insulin (18.4%). Female gender, pubertal age and poor glycemic control were associated with higher and migration background with lower total costs.

**Conclusions:**

Main cost categories in patients with on average 11 years of diabetes duration were costs for glucose self-monitoring, insulin pump therapy, hospitalization and insulin. Optimization of glycemic control in particular in pubertal age through intensified care with improved diabetes education and tailored insulin regimen, can contribute to the reduction of direct diabetes-related costs in this patient group.

## Introduction

Pediatric type 1 diabetes is an illness with severe and long-lasting impact for the individual and its family, and in addition for the society [1], [2]. In particular, children with early-onset pediatric type 1 diabetes show a more severe disease onset with increased risk of diabetic ketoacidosis [3], [4]. They also bear greater life-time risks to develop diabetes-related chronic complications as a consequence of their almost life-long diabetes duration [5]. According to the German CoDiM Study, direct diabetes-related costs increase considerably with the onset of diabetes-related complications [6], [7].

By now, there is limited information on the costs of pediatric type 1 diabetes. Only a few studies discuss direct diabetes-related costs of pediatric diabetes [8]–[11] with a wide range of estimated costs (about €2,800–6,200 per person-year).

So far, the studies analyzing diabetes-related costs mainly focused on mean costs of diabetes in patients with relatively short duration and irrespective of the age at onset. There are no specific studies on direct medical cost in early-onset pediatric type 1 diabetes of long duration. However, in times of limited economic resources it is essential to keep the health care resources in mind. This issue will gain importance in pediatric diabetes care since pediatric type 1 diabetes is increasing in Europe as well as worldwide [12]–[15]. A rapid incidence increase was particularly observed in children younger than five years of age and the number of new cases in this age group has been predicted to double between 2003 and 2020 [12].

The aim of this study was to estimate direct diabetes-related health care costs of pediatric type 1 diabetes and to assess their predictors in a population-based cohort of young patients with early-onset diabetes and at least eight years of diabetes duration.

## Research Design and Methods

### Study population

The study cohort was selected from the nationwide diabetes register maintained at the German Diabetes Center (DDZ) since 1993 [16]. Completeness of ascertainment of the register is estimated at about 95%. Basic inclusion criterion for this analysis was an onset of type 1 diabetes during the first five years of life between the years 1993 and 1999. Overall, 3,224 registered cases met the inclusion criteria. This cohort is followed-up within a project of the German Competence Network Diabetes Mellitus (www.kompetenznetz-diabetes-mellitus.net/index.php/en). Data on health care utilization in 2007 were taken from the DPV (Diabetes Software for Prospective Documentation) database and used as basis for estimating direct diabetes-related health care costs. DPV is a trusted, well-established software for prospective longitudinal documentation of routine diabetes care and outcomes [17]–[20]. It is presently used in more than 300 pediatric diabetes centers in Germany, which is the majority of German facilities with pediatric diabetes care. Twice yearly, data of all treatment centers are anonymized and transmitted to the Institute of Epidemiology and Medical Biometry at Ulm University for central plausibility checks. Inconsistent data are reported back to participating centers for validation and correction. Finally, corrected and validated data are jointly entered into a cumulative database for further analyses. To ensure that patients were treated continually, only subjects who had at least a once annual documentation in DPV for the years 2006 to 2008 were included. Thus, health care utilization is assumed to be largely covered for these patients. Altogether, data of 1,473 subjects (45.7% of eligible subjects in Germany) from 199 German diabetes care centers were available for analysis. The analyses were based on data for the year 2007.

### Assessment of sociodemographic and clinical variables

Information about gender, age (as of 31 December 2007), and diabetes duration was available for all subjects. Migration background was assumed, if a subject's father or mother or both were born outside of Germany. For analysis of glycemic control, individual mean HbA1c in 2007 was standardized to the Diabetes Control and Complications Trial (DCCT) normal range by means of the “multiple of the mean method” [21]–[24]. In doing so, it was accounted for differences between center laboratories in HbA1c due to differing methods of measurement. HbA1c values were finally categorized according to recommendations of the International Society for Pediatric & Adolescent Diabetes (ISPAD) (<58, 58-<75, ≥75 mmol/mol, corresponding to <7.5, 7.5-<9.0; ≥9%) [25]. All these factors have previously been reported to be associated with costs of care [9], [11], [26]–[28].

### Health care utilization

Health care service utilization included the number, the reason for and the length of hospital stays related to diabetes, the frequency of diabetes-related outpatient consultations (routine medical services and laboratory testing), the type of insulin and the insulin regimen, the mean daily insulin dose (international units, IU), and the number of daily blood glucose self-measurements (median of all documented values in 2007) for each subject. Additionally, the treatment with antihypertensive drugs (ACE inhibitors), lipid-lowering agents (statins), and biguanides (metformin) was included.

### Cost data

In Germany, the vast majority of the population (approximately 85% [29]) is insured via the statutory health insurance and almost all medical costs for diabetes care are covered by the health insurance. For this reason, the study took the perspective of the statutory health insurance. Since children and adolescents in Germany have free access to services in the health care sector, no patients' co-payments were included. Prices for insulin and other medication were taken from the official German Index of Medicines of 2007 by calculating mean retail prices for the biggest privately available package-size of the year 2007. For insulin pumps, syringes, lancets, blood glucose and ketone body strips as well as glucagon sets prices for 2007 were estimated by deflating 2010 prices using the Consumer Price Index (www.destatis.de). In Germany, between 2003 and 2005, diagnosis-related groups (DRGs) were implemented as a reimbursement system for inpatient care. Within the DRG system, hospitalizations are reimbursed by fixed rates mainly depending on diagnosis (www.g-drg.de). In table 1, average health care utilization of the study group is shown. Furthermore, there are corresponding unit costs for all included health care services. Fees for outpatient medical service were taken from the statutory health insurances' price scale (per capita quarterly rates plus additional fees for subjects undergoing laboratory testing). For subjects registered in a structured healthcare program for chronically ill patients (Disease Management Program [DMP]), average DMP fees were applied for outpatient care instead.

**Table 1:** Health care utilization included in cost analysis, and unit costs [€, 2007].

Units	Mean units in study population	Unit costs [€]	Assumptions
Hospital admission^a^ per patient	0.35	2,393 per hospital admission	
Outpatient care^b^: consultations per person	4.2	30.91 quarter-year rate for patients >5 years (additionally 1.28 for younger children);	routine diabetes-related medical services including laboratory testing
		In case of Disease Management Program participation 24.91 for the first quarter-year with DMP documentation, afterwards 15.66 for each quarter-year with documentation plus quarter-year rate 39.27 for outpatient care	
		additional fees for laboratory tests (0.25 per blood glucose measurement, 4.0 per HbA1c measurement, 0.25 per annual cholesterol measurement)	
Mean (SD) insulin dose (IU)^c^ per patient per day	49.7 (19.2)	0.03 per IU of regular insulin0.04 per IU of short-acting insulin analogues0.05 per IU of long-acting insulin analogues	
Injection needles/syringes per patient per day	0.674	0.26 per needle/syringe^§^	use of one syringe/injection needle per day, free provision of pens by the pharmaceutical industry
Insulin pumps per patient	0.362	3490.41 per insulin pump^d^	projected durability of an insulin pump was four years
Pump catheter per patient per day	0.163	10.43 per syringe^d^^¶^	one pump infusion set every two days
Mean (SD) number of blood glucose measurements per patient per day	5.1 (1.5)	0.55 per strip^d^	one blood glucose self-testing strip per measurement, free provision of glucometers by the pharmaceutical industry
Lancets per patient per day	1	0.11 per lancet^d^	one lancet per day
Urine ketone self-measurement package per patient	1	12.22 per package^d^	one package of urine ketone strips per patient per year
Glucagon sets per patient	1	29.25 per set^d^	one glucagon set per patient per year
Daily dose of ACE inhibitors^c^ per patient	0.017	0.20 per daily dose	daily dose of 10 mg in case of antihypertensive treatment
Daily dose of lipid-lowering agents^c^ (statins) per patient	0.002	0.46 per daily dose	daily dose of 20 mg
Daily dose of biguanides^c^ per patient (metformin)	0.003	0.13 per daily dose	daily dose of 850 mg

a Reference: patient-specific, German diagnosis-related groups (website of the Institute for the Hospital Remuneration System. Available: http://www.g-drg.de. Accessed 2011Jun 8).

b Reference: uniform value scale of the German health insurance system (EBM); DMP: mean value from German DMPs.

c Reference: Rote Liste 2007, i. e. the German equivalent of the Physicians' Desk Reference.

d Mean price of the year 2010, deflated to 2007 using the Consumer Price Index (website of the Federal Statistical Office. Available: http://www.destatis.de. Accessed 2011 Dec 5).

### Statistical analyses

For all continuous variables, descriptive statistics with mean values, standard deviations (SD) and ranges were calculated. For categorical variables, proportions of subjects were used for description. In accordance with Thompson [30], total costs and costs in different subcategories were expressed as mean costs per patient-year in Euro (€) along with inter-quartile ranges.

Mean total costs, mean costs for self-monitoring of blood and urine glucose (SMBUG), or for insulin were dependent variables. Gender, age (8–11, 12–15, 16–19 years, as of 31 December 2007), diabetes duration (8–10, 11–14 years, as of 31 December 2007), migration background, and glycemic control (HbA1c <58, 58-<75, ≥75 mmol/mol, corresponding to <7.5, 7.5-<9.0, ≥9%) constituted independent variables. The associations between independent and dependent variables were analyzed using multiple log-linear regression models (Ordinary Least Squares estimation for log-transformed costs) [31], [32]. Cost ratios were estimated by retransformation of expected cost differences on the log-scale. Estimates of expected costs were derived from regression coefficients using the non-parametric Smearing transformation [32]. Because most subjects had no inpatient treatment or no continuous subcutaneous insulin infusion (CSII) therapy in 2007, two-part models were applied to analyze determinants of costs for hospitalization or CSII in the total cohort [8], [31]–[33]. First, the relative risk (RR) for hospitalization or CSII therapy was estimated by multiple log-binomial regression [33]. Second, a multiple log-linear regression procedure was used to assess expected cost ratios among subjects with inpatient care or CSII therapy. The convenience of applying log-binomial regression in the first part of the model is that results from both parts of the model can easily be combined to estimate expected cost ratios and expected costs for the whole study population (unlike for the common logistic approach). Total cost ratios were then estimated by multiplying the relative risk of hospitalization or CSII therapy and the expected cost ratios of hospitalized subjects or subjects with CSII therapy, respectively [32]. Accordingly, total expected costs were estimated by multiplying the probability of hospitalization or CSII therapy and the expected costs of hospitalized subjects or subjects with CSII therapy, respectively [32]. All tests were performed at a two-sided significance level of 0.05. For statistical evaluation, SAS for Windows Version 9.2 (SAS Institute, Cary, NC, USA) was used.

The study was approved by the ethical review board of Heinrich Heine University Düsseldorf.

## Results

### Study population

Table 2 describes the characteristics of the patients. Mean age was 13.9 (SD 2.2) years, mean diabetes duration was 10.9 (SD 1.9) years. A total of 21.7% of the study patients were 8–11 years old, 58.2% were 12–15 years old, and 20.0% were 16–19 years old. Altogether 53.8% of the patients had a diabetes duration of 8–10 years and 46.2% of 11–14 years.

**Table 2:** Description of the study population 2007.

	Study population
N	1,473
Boys	780 (53.0%)
Age as of 31 December 2007 [years]; mean ± SD (range)	13.92±2.21 (8–19)
Diabetes duration as of December 31, 2007 [years]; mean ± SD (range)	10.94±1.93 (8–14)
Patients with migration background	202 (13.7%)
HbA1c [mmol/mol] ([%]), mean ± SD	64.6±15.6 (8.06±1.43)
<58 mmol/mol (<7.5%)	607 (41.4%)
58-<75 mmol/mol (7.5-<9%)	560 (38.1%)
≥75 mmol/mol (≥9%)	301 (20.5%)
Patients using insulin pumps	533 (36.2%)
continuously in 2007	388 (26.3%)
starting CSII in 2007	145 (9.8%)
Patients prescribed ACE-inhibitors	25 (1.7%)
Patients prescribed lipid-lowering agents (statins)	4 (0.3%)
Patients prescribed biguanides (Metformin)	3 (0.2%)
Number of outpatient consultations	6,169
Patients with ≥1 consultation each quarter-year	731 (49.6%)
Patients with no consultation	36 (2.4%)
Patients enrolled in a Disease Management Program (DMP)	397 (36.9%)
Number of diabetes-associated hospital admissions (hospital days) in # patients	509 (4194) (422)
Patients with 1 admission	362 (24.6%)
Patients with 2 admissions	41 (2.8%)
Patients with >2 admissions	19 (1.3%)
Diabetes-related hospital inpatient days per patient in patients with hospital admission, mean ± SD	8.81±11.86
Patients with hospital stays by length	
No hospital stay	1,051 (71.4%)
1–7 days	263 (17.9%)
8–14 days	93 (6.3%)
15–21 days	17 (1.2%)
>21 days	49 (3.3%)
Reasons for hospitalization [% of stays (days)]	
diabetes manifestation	-
metabolic adjustment/diabetes education	72.5% (78.5%)
acute hyperglycaemia	15.9% (14.1%)
routine care	4.3% (1.0%)
acute hypoglycemia	4.3% (2.2%)
late complications	2.8% (4.0%)

### Utilization of diabetes care

More than one third of the subjects (36.2%) were on CSII therapy, nearly three-quarters of those (73.0%) used insulin pump therapy throughout 2007, the others started CSII therapy in 2007. Mean insulin dose per kilogram body weight was 0.9 (SD 0.3) IU. Antihypertensive drugs were taken by 1.7%, lipid-lowering agents by 0.3%, and biguanides by 0.2% of the subjects, respectively.

Regarding outpatient consultations, half of the subjects had at least one contact per quarter-year (49.6%). The mean number of diabetes-related outpatient consultations was 4.2 per year. More than a third of the subjects (36.9%) were registered in a DMP.

During 2007, most of the subjects (71.4%) had no inpatient treatment because of diabetes. A quarter of the study patients had one stay (24.6%), 4.1% had more than one diabetes-related hospitalization. The average hospital stay lasted 8.8 days. In 2007, a total of 17.9% of the patients had a cumulative length of inpatient treatment of up to one week, 6.3% of >1–2 weeks, 1.2% of >2–3 weeks, and 3.3% of more than 3 weeks. Further details on the reasons for diabetes-related hospital admissions and days in hospital are reported in table 2. Related to the whole study population, there was a mean of 0.35 diabetes-related hospital stays and 2.85 hospital days per person-year in 2007.

### Factors associated with inpatient care or CSII therapy

Table 3 summarizes the results of the first part of the applied two-part model and gives an overview of factors that were associated with hospitalization and CSII therapy. The risk for hospitalization was significantly lower in 16 to 19-year-olds compared with 8–11 year old patients (RR 0.67, 95% confidence interval (95%-CI) 0.48–0.94). Impaired and poor glycemic control were associated with significantly higher hospitalization (RR 1.60, 95%-CI 1.30–1.96 and 2.31, 95%-CI 1.87–2.86, respectively). Gender, diabetes duration, and migration background were not significantly associated with hospitalization (table 3). The use of CSII therapy was significantly lower among male patients (RR 0.76, 95%-CI 0.67–0.87) and patients with migration background (RR 0.72, 95%-CI 0.57–0.92). Age, diabetes duration, and glycemic control were not associated with CSII (table 3).

**Table 3:** Factors associated with inpatient care and CSII therapy.

	Relative risk for hospitalization	Relative risk for CSII therapy
	(95%-CI)^a^	(95%-CI)^a^
Gender		
Male vs. female	1.07 (0.92,1.26)	0.76 (0.67,0.87)^b^
Age (ref. 8–11 years)		
12–15 years	1.03 (0.83,1.29)	1.20 (0.98,1.47)
16–19 years	0.67 (0.48,0.94)^b^	1.10 (0.83,1.45)
Migration background		
yes vs. no	1.03 (0.83,1.27)	0.72 (0.57,0.92)^b^
Diabetes duration		
11–14 vs. 8–10 years	0.99 (0.82,1.20)	1.13 (0.95,1.34)
HbA1c (ref. <58 mmol/mol (<7.5%))		
58- <75 mmol/mol (7.5-<9.0%)	1.60 (1.30,1.96)^b^	1.01 (0.88,1.17)
≥75 mmol/mol (≥9%)	2.31 (1.87,2.86)^b^	0.85 (0.70,1.03)

a Estimates of relative risks are derived from multiple log-binomial regression models including all factors.

b p<0.05.

### Diabetes-related costs

In 2007, average direct diabetes-related health care costs per study subject amounted to €3,745 (table 4). The spread of individual total costs among study patients was considerable, ranging from €948 up to €34,498 (inter-quartile range: €1,943–4,881). The largest share of costs in the study cohort was attributable to SMBUG, followed by CSII and diabetes-related hospitalizations. Almost one fifth of the costs were spent for insulin. A total of 3.6% was spent for diabetes-related outpatient care. The percentage of diabetes-related costs for injection needles/syringes, glucagon sets, and other medication amounted to each at most 2% of total costs.

**Table 4:** Diabetes-related costs per patient in the study population (age group 8–18 years, onset 1993–1999 at 0–4 years) in 2007.

	Costs per patient in the study population 2007 [€]: mean (inter-quartile range)	Proportion of annual costs [%]
Diabetes-related hospitalization (n = 422)	827 (0–1,862)	22.1
Diabetes-related outpatient care (n = 1,437)	134 (106–142)	3.6
Insulin	689 (473–859)	18.4
Self-control (blood/urine glucose self-measurement strips, lancets)	1,066 (855–1,212)	28.5
Injection needles/syringes	64 (0–95)	1.7
CSII pump and pump infusion sets (n = 533)	936 (0–2,776)	25.0
Glucagon sets	29^a^	0.781
Antihypertensive drugs (n = 25)	0.81^b^	0.022
Lipid-lowering drugs (n = 4)	0.09^b^	0.002
Biguanides (n = 3)	0.14^b^	0.004
Total annual costs	3,745 (1,943–4,881)	100

a no IQR because of fixed estimation (one needle per patient per day and one set per patient per year).

b no IQR because of small user number.

### Factors associated with diabetes-related costs

Associations between total costs or different cost categories and influencing factors are given in table 5 in terms of expected cost ratios and in table 6 in terms of expected costs and cost differences.

**Table 5:** Factors associated with total direct medical costs and different cost categories per patient: Estimates of cost ratios^a^.

	Total direct medical costs	Costs for hospitalization	Costs for insulin	Costs for self-monitoring of blood and urine glucose	Costs for CSII therapy
	Cost ratio (95%-CI)	Cost ratio (95%-CI)	Cost ratio (95%-CI)	Cost ratio (95%-CI)	Cost ratio (95%-CI)
Gender					
Male vs. female	0.94 (0.89,0.99)^b^	1.08 (0.89,1.31)	0.98 (0.94,1.01)	1.01 (0.98,1.03)	0.74 (0.64,0.85)^b^
Age (ref. 8–11 years)					
12–15 years	1.10 (1.02,1.19)^b^	1.02 (0.78,1.34)	1.54 (1.47,1.62)^b^	0.93 (0.90,0.96)^b^	1.22 (0.98,1.52)
16–19 years	1.00 (0.89,1.12)	0.55 (0.37,0.83)^b^	1.78 (1.65,1.91)^b^	0.84 (0.80,0.89)^b^	1.14 (0.85,1.54)
Migration background					
Yes vs. no	0.92 (0.84,0.99)^b^	1.12 (0.86,1.46)	0.97 (0.92,1.02)	0.94 (0.90,0.98)^b^	0.65 (0.50,0.84)^b^
Diabetes duration					
11–14 vs. 8–10 years	1.05 (0.97,1.13)	0.95 (0.75,1.2)	1.15 (1.10,1.20)^b^	0.96 (0..93,0.99)^b^	1.16 (0.97,1.39)
HbA1c					
(ref. <58 mmol/mol (<7.5%))					
58-<75 mmol/mol (7.5-<9.0%)	1.08 (1.02,1.15)^b^	1.91 (1.50,2.44)^b^	1.08 (1.04,1.12)^b^	0.92 (0.90,0.95)^b^	0.99 (0.85,1.16)
≥75 mmol/mol (≥9%)	1.14 (1.05,1.23)^b^	2.99 (2.31,3.87)^b^	1.12 (1.07,1.17)^b^	0.82 (0.79,0.85)^b^	0.88 (0.71,1.08)

a Estimates of cost ratios are derived by retransformation of estimates from multiple log-linear regression models or two-part models (hospitalization, CSII) including all factors.

b p<0.05.

**Table 6:** Factors associated with total direct medical costs and different cost categories per patient: Estimates of cost and cost differences^a^.

	Total direct medical costs [€]	Costs for hospitalization [€]	Costs for insulin [€]	Costs for self-monitoring of blood and urine glucose [€]	Costs for CSII therapy [€]
	(95%-CI)	(95%-CI)	(95%-CI)	(95%-CI)	(95%-CI)
Gender					
Female	3878 (3719,4036)	796 (676,917)	699 (681,717)	1062 (1042,1082)	1084 (979,1190)
Male	3638 (3497,3779)	860 (741,979)	683 (666,699)	1070 (1051,1089)	810 (719,901)
Diff. Male - Female	−240 (−453,−27)^b^	64 (−106,234)	−16 (−40,8)	8 (−20,35)	−274 (−414, −135)^b^
Age					
8–11 years	3545 (3297,3794)	903 (668,1137)	466 (445,486)	1149 (1112,1185)	817 (652,983)
12–15 years	3902 (3758,4047)	924 (808,1040)	716 (700,733)	1067 (1049,1085)	988 (986,1080)
16–19 years	3532 (3272,3792)	499 (347,651)	827 (790,864)	969 (935,1002)	918 (747,1090)
Diff. ‘12–15’ - ‘8–11’	357 (74,640)^b^	22 (−239,282)	251 (225,276)^b^	−82 (−122, −41)^b^	171 (−18,360)
Diff. ‘16–19’ - ‘8–11’	−13 (−418,391)	−404 (−708, −99)^b^	361 (314,408)^b^	−180 (−235, −125)^b^	101 (−163,365)
Migration background					
No	3795 (3680,3911)	814 (723,906)	693 (680,706)	1075 (1060,1090)	981 (905,10560)
Yes	3474 (3208,3740)	913 (678,1149)	673 (641,705)	1010 (974,1045)	662 (494,830)
Diff. yes – no	−321 (−612, −30)^b^	99 (−155,353)	−20 (−55,14)	−66 (−104, −27)^b^	−319 (−503, −134)^b^
Diabetes duration					
8–10 years	3672 (3509,3835)	847 (720,974)	640 (622,659)	1085 (1063,1106)	879 (775,984)
11–14 years	3841 (3655,4027)	808 (666,949)	736 (716,757)	1043 (1019,1067)	1004 (885,1124)
Diff. ‘11–14’ - ‘8–10’	169 (−109, 447)	−40 (247,168)	96 (64,127)^b^	−42 (−78, −5)^b^	125(−51,301)
HbA1c					
<58 mmol/mol (<7.5%)	3538 (3382,3695)	477 (378,575)	653 (635,671)	1140 (1117,1163)	965 (856,1075)
58-7<5 mmol/mol (7.5-<9.0%)	3830 (3656,4005)	912 (765,1058)	705 (685,726)	1052 (1030,1074)	966 (855,1077)
≥75 mmol/mol (≥9%)	4028 (3772,4283)	1426 (1169,1682)	731 (703,759)	938 (911,966)	832 (681,983)
Diff. ‘58-<75 mmol/mol - <58 mmol/mol’ ‘ (7.5-<9.0%’ – ‘<7.5%’)	292 (58,525)*	435 (259.612)*	52 (26,79)*	−88 (−120, −56)*	0 (−155,156)
Diff. ‘≥75 mmol/mol/- <58 mmol/mol’ (‘≥9%’ – ‘<7.5%’)	489 (187,791)*	949 (672,1226)*	78 (44,112)*	−202 (−238, −166)*	−133 (−321,55)

a Estimates of cost and cost differences are derived by applying the Smearing retransformation to estimates from multiple log-linear regression models or two-part models (hospitalization, CSII) including all factors.

b p<0.05.

Total costs and costs for CSII therapy in male patients were 6.2% (€240) and 26% (€274) lower than in female patients, respectively. Reduced CSII costs among the male subgroup were mainly attributable to the reduced rate of CSII therapy in this group (table 3). There were no significant gender differences with respect to the other single cost categories. Total costs, costs for hospitalizations, and CSII costs were highest in the age group of the 12 to 15-year-olds. In contrast, costs for insulin increased steadily and costs for SMBUG dropped steadily with increasing age. Patients with a diabetes duration of 11–14 years had 15% (€96) higher costs for insulin, but 4.1% (€42) lower costs for SMBUG compared with patients with 8–10 years of diabetes duration. Poor glycemic control was significantly associated with increased total costs, higher costs for hospitalization (up to three times higher costs) and higher costs for insulin, but with decreased costs for SMBUG. Glycemic control was not significantly associated with costs for CSII therapy. Patients with migration background had significantly lower total costs and lower costs for SMBUG and CSII therapy, but higher costs for hospitalization (not statistically significant).

## Conclusions

### Main findings and implications

Using a population-based cohort and data of a standardized prospective computer-based documentation system (DPV), we estimated direct diabetes-related health care costs of a special group of patients with early disease onset and at least 8 years of diabetes duration, who were expected to have increased total direct diabetes-related costs compared with patients with shorter diabetes duration. Average costs of subjects with early-onset and long diabetes duration amounted to €3,745 per patient-year. Costs varied widely between different patient groups. As in previous cost analyses [11], [34], total diabetes-related costs were lower in male than in female patients. Worse glycemic conditions in pubertal age [18], [35], [36] and higher comorbidity [37], [38] or more intensive health care seeking behavior in female subjects [39] may explain these findings. There was a strong association between diabetes-related costs and HbA1c as an indicator for glycemic control. Worse glycemic control was associated with increased total costs, costs for hospitalization, and costs for insulin. We hypothesize that subjects with worse glycemic control measure their blood glucose level less frequently, but have more hospitalizations and higher demands for insulin. The decrease with age observed in the costs for SMBUG corresponds to less frequent glycemic control with older age as previously shown [40]. Migration background was associated with lower total direct medical costs. This was obviously due to less utilization of SMBUG and in particular CSII therapy (tables 3 and 5) in patients with migration background.

### Comparison of costs in the study population to average costs in pediatric patients in Germany

Despite almost two-fold longer diabetes duration, direct diabetes-related health care costs were only 6.3% higher than estimated average costs in previously analyzed patients in Germany (€3,524), who were slightly younger (mean age 12.1 (SD 4.2) years) and had about half the diabetes duration (mean duration 4.7 (SD 3.6) years) [8]. This can be attributed to the fact that the study patients with at least 8 years of diabetes duration have largely remained free of severe chronic diabetic complications, as is indicated by the low rate of inpatient care because of complications and the lack of medication for comorbidities. The German Costs of Diabetes Mellitus (CoDiM) study revealed that the treatment of diabetic complications was far more costly than the underlying disease [7]. Therefore, the analysis of patients with diabetes duration longer than ten years on average is likely to reveal different results. In former analyses, mean diabetes duration of DPV patients with severe diabetes-related retinopathy was 26.5 years [41] and the proportion of patients affected by renal complications increased with age [17]. Other cohort studies corroborate these findings. For example, in case of renal complications, data from the Swedish Childhood Diabetes Registry (SCDR) [42], the Epidemiology of Diabetes Interventions and Complications (EDIC) study [43], [44], and the Allegheny County population-based registry [45] show that severe complications can be expected much later than after one decade of disease duration.

Absolute and relative costs for insulin and CSII were higher in study patients with long diabetes duration compared with a previously analyzed pediatric cohort of DPV patients <20 years of age with on average half the diabetes duration [8]. The reason for an increase in insulin requirement may be due to insulin resistance with increasing age and diabetes duration. Furthermore, the honeymoon period, a period after the initial treatment of diabetes, in which the insulin production temporarily regenerates, reduces average insulin requirement in the previously analyzed group of pediatric patients with type 1 diabetes <20 years of age. The increase in CSII costs is attributable to increased use of CSII therapy in patients with longer diabetes duration and thus more complicated glycemic control. In contrast, patients of this study cohort had lower absolute and relative costs regarding SMBUG, hospitalization, and outpatient care compared with the previously analyzed cohort with shorter diabetes duration. Further investigation should consider whether the present findings will apply to patients with even longer diabetes duration.

### Comparison of the study results with previous reports

In general, it's difficult to compare findings of studies because there is large heterogeneity regarding methodological approaches, but also with respect to patients included (type 1, 2 or both, age classes, diabetes duration), and reimbursements under different national health care systems. Most studies analyzed patients irrespective of diabetes duration. Therefore, comparability with the present results is limited. In particular, results of studies including patients with type 2 diabetes are not comparable due to differences in therapeutic regimes (e. g. number of prescribed test strips, prescription of insulin and/or oral antidiabetic medication) and resulting costs [10], [11], [46], [47].

In a recent examination in the U.S., Ying et al. estimated direct costs of diabetes care in type 1 diabetic subjects less than 20 years of age (mean age 12.8 (SD 3.7) years) [9]. Mean total diabetes-related direct costs per person-year were US$4,730 (i.e. €3,493) and thus were slightly lower than our estimates. Mean diabetes duration of the subjects of Ying et al. was 5.6 (SD 3.1) years in contrast to 10.9 years in the present cohort. However, as patients with diabetes duration less than 6 months were excluded by Ying et al., a comparison seems feasible. Main cost categories in the study of Ying et al. were supplies (38% of total annual costs), medication (33%), and hospitalization (15%). Other cost categories were outpatient visits (9%), emergency room (4%), and education visits (1%). In our study, main contributors to total costs were the same, but the share of single cost items was different, probably due to differences in health care systems and diabetes therapy between the U.S. and Germany. Furthermore, the cost categories used by Ying et al. are different from ours, so that a direct comparison is difficult. CSII therapy, for example, is not separately listed by Ying et al.

Other studies reported lower direct medical costs in subjects with type 1 diabetes in Australia (AU$3640 (i.e. €2,283), mean age 32.0 (range 5–88) years, mean diabetes duration 8.3 years) [47] and Spain (€2,104, mean age 29.2 (SD 12.2), mean diabetes duration 10.5 (SD 7.9) years) [26].The subjects of the Spanish study had lower costs for hospitalization (€164 vs. €827) [26]. In Germany, a large proportion of diabetes education is commenced in the hospital setting and this is likely to be one reason for high hospitalization costs. Unfortunately, Ballesta et al. [26] did not report whether costs for structured patient education were covered by their analysis. Furthermore, Spanish subjects measured blood glucose less frequently than German ones (2.7 vs. 5.1 times per day) and costs for CSII were not included.

### Study limitations and strength

Despite the advantage of including a large population-based cohort of type 1 diabetic patients and the benefits of continuous standardized documentation from a comprehensive computer database, some limitations have to be considered.

The study cohort was selected from a population-based, almost complete incidence register. We could include nearly 50% of all patients who were diagnosed between the years 1993–1999. The proportion of males in study patients was comparable to that among patients not included (53.0% vs. 52.0%). On average, study patients were slightly younger and had a slightly shorter diabetes duration than not included patients (mean age 15.04 (SD 2.50) years, mean diabetes duration 11.80 (SD 2.07) years). Further, we cannot exclude that our study group differs from not included patients with respect to other potentially influencing factors, e.g. education, household income or socio-economic status, as respective information was not available. Thus, a selection bias cannot be excluded, even though DPV is used in a broad spectrum of pediatric facilities including small-sized centers and tertiary university centers. Our inclusion criteria (at least one annual documentation during 2006–2008) may have caused further selection bias by excluding relatively non-adherent patients who would generate more or less diabetes-related costs. Although diabetes care is highly standardized in Germany and available for all patients and covered by the statutory health insurance, the results may not apply to all respective patients with type 1 diabetes in Germany. Nevertheless, we consider our results to hold true for most patients with early-onset type 1 diabetes and more than 8 years of diabetes duration according to the inclusion criteria of the study.

Total cost of health care may not be transferable to other countries due to the varying basic conditions of health care systems. However, the estimated cost ratios can be assumed to be less dependent on such basic conditions and thus to be better comparable to other countries (table 5).

The DPV patient documentation is used by many clinics for administrative purposes (basis for reimbursement of costs from statutory health insurance funds), and regular checks on plausibility and validity of data are implemented. Therefore, the data of health care utilization are supposed to be rather valid. However, some extent of underreporting cannot completely be excluded. Further, we cannot completely exclude that patients have received diabetes care outside the DPV coverage. Thus, health care utilization may be underestimated in this study.

Regarding outpatient care, we employed the general items of the medical fee schedule. For subjects in DMP, only fees for basic diabetes care were considered, as compensation for further health care services varies enormously among the different DMPs. In addition, special diabetes-related per capita fees were not considered in our estimations. Thus, our cost estimates are expected to be rather conservative.

As prices for insulin and other medication were derived from the official German Index of Medicines by calculating mean retail prices for the biggest privately available package-size, respective cost estimates are assumed to be conservative and lower bound of real costs.

We used 2010 mean prices for insulin pumps, syringes, lancets, blood glucose and ketone body strips as well as for glucagon sets to estimate 2007 prices. Since we deflated medical prices of the year 2010 to 2007, we consider our cost estimates to be valid.

In conclusion, type 1 diabetes in young patients with early disease onset and about 11 years of diabetes duration is an economic challenge, even before the development of severe and costly complications related to diabetes. Main cost categories were costs for glucose self-monitoring, pump therapy, hospitalization, and insulin. Glycemic control and pubertal age were main determinants for total costs, while diabetes duration had little effect on total costs at this disease stage. Optimization of glycemic control and intensified care, in particular in pubertal age, can contribute to the reduction of direct diabetes-related costs. Further analyses should estimate costs of patients with longer diabetes duration.

## Supporting Information

Appendix S1 DPV centers contributing data for this analysis.
(DOCX)
