# Supplementary material for: Direct Diabetes-Related Costs in Young Patients with Early-Onset, Long-Lasting Type 1 Diabetes
Source: PLoS One. 2013 Aug 13;8(8):e70567. doi: 10.1371/journal.pone.0070567 (PMC3742743; doi:10.1371/journal.pone.0070567)
Supplement: Appendix S1 — DPV centers contributing data for this analysis. (DOCX) [file pone.0070567.s001.docx]

## DPV centers contributing data for this analysis

Aachen - Uni-Kinderklinik RWTH, Aalen Kinderklinik, Arnsberg-Hüsten Karolinenhospital, Aue Helios Kinderklink, Augsburg Kinderklinik Zentralklinikum, Aurich Kinderklinik, Bad Aibling Internist. Praxis, Bad Hersfeld Kinderklinik, Bad Kösen Kinder-Rehaklinik, Bad Lauterberg Diabeteszentrum, Bad Mergentheim – Diabetesfachklinik, Bad Mergentheim - Gemeinschaftspraxis DM, Bad Oeynhausen Herz-und Diabeteszentrum, Bad Orb Spessart Klinik, Bad Salzungen Kinderklinik, Bad Waldsee Kinderarztpraxis, Bautzen Oberlausitz Kinderklinik, Berchtesgaden CJD, Berlin DRK-Kliniken, Berlin Endokrinologikum, Berlin Kinderklinik Lindenhof, Sana Klinik, Berlin Virchow-Kinderklinik, Bielefeld Kinderklinik Gilead, Bocholt Kinderklinik, Bochum Universitätskinderklinik St. Josefskrankenhaus, Bonn Uni-Kinderklinik, Bottrop Kinderklinik, Braunschweig Kinderarztpraxis, Bremen - Kinderklinik Nord, Bremen – Mitte Innere Medizin, Bremen Kinderklinik St.Jürgenstraße, Bremerhaven Kinderklinik, Celle Kinderklinik, Chemnitz Kinderklinik, Coesfeld Kinderklinik, Darmstadt Kinderklinik Prinz. Margaret, Datteln Vestische Kinderklinik, Delmenhorst Kinderklinik, Detmold Kinderklinik, Dortmund Kinderklinik, Dortmund Knappschaftskrankenhaus, Dortmund Medizinische Kliniken Nord, Dresden Neustadt Kinderklinik, Dresden Uni-Kinderklinik, Düren-Birkesdorf Kinderklinik, Düsseldorf Uni-Kinderklinik, Erfurt Kinderklinik, Erlangen Uni-Kinderklinik, Essen Diabetes-Schwerpunktpraxis, Essen Elisabeth Kinderklinik, Essen Uni-Kinderklinik, Eutin Kinderklinik, Forchheim Diabeteszentrum SPP, Frankfurt Uni-Kinderklinik, Freiburg Uni-Kinderklinik, Friedrichshafen Kinderklinik, Fulda Innere Medizin, Fulda Kinderklinik, Fürth Kinderklinik, Gaissach Fachklinik der Deutschen Rentenversicherung, Garmisch-Partenkirchen Kinderklinik, Gelsenkirchen Kinderklinik Marienhospital, Gießen Uni-Kinderklinik, Göppingen Kinderklinik am Eichert, Görlitz Städtische Kinderklinik, Göttingen Uni-Kinderklinik, Hagen Kinderklinik, Halle Uni-Kinderklinik, Hamburg Altonaer Kinderklinik, Hamburg Endokrinologikum, Hamburg Kinderklinik Wilhelmstift, Hamburg-Nord Kinder-MVZ, Hamm Kinderklinik, Hanau Kinderklinik, Hannover Kinderklinik MHH, Hannover Kinderklinik auf der Bult, Haren Kinderarztpraxis, Heide Kinderklinik, Heidelberg Uni-Kinderklinik, Heidenheim Kinderklinik, Heilbronn Kinderklinik, Herdecke Kinderklinik, Herford Kinderarztpraxis, Herford Klinikum Kinder & Jugendliche, Heringsdorf Inselklinik, Hermeskeil Kinderpraxis, Herne Evangelisches Krankenhaus, Herten St. Elisabeth, Hildesheim Innere Medizin, Hildesheim Kinderarztpraxis, Hildesheim Klinikum Kinderklinik, Hinrichssegen-Bruckmühl Diabetikerjugendhaus, Homburg Uni-Kinderklinik Saarland, Iserlohn Innere Medizin, Itzehoe Kinderklinik, Jena Uni-Kinderklinik, Kaiserslautern-Westpfalzklinikum Kinderklinik, Karlsburg Klinik für Diabetes & Stoffwechsel, Karlsruhe Städtische Kinderklinik, Kassel Kinderklinik Park Schönfeld, Kassel Städtische Kinderklinik, Kaufbeuren Innere Medizin, Kempen Heilig Geist, Kiel Städtische Kinderklinik, Kiel Universitäts-Kinderklinik, Kirchen DRK Klinikum Westerwald, Kinderklinik, Koblenz Kinderklinik Kemperhof, Konstanz Kinderklinik, Krefeld Kinderklinik, Kreischa-Zscheckwitz Klinik Bavaria, Köln Kinderklinik Amsterdamerstraße, Köln Uni-Kinderklinik, Landshut Kinderklink, Leipzig Uni-Kinderklinik, Leverkusen Kinderklinik, Lingen Kinderklinik St. Bonifatius, Lippstadt Evangelische Kinderklinik, Ludwigsburg Kinderklinik, Ludwigshafen Kinderklinik St. Anna-Stift, Lübeck Uni-Kinderklinik, Lüdenscheid Kinderklinik, Magdeburg Uni-Kinderklinik, Mainz Uni-Kinderklinik, Mannheim Uni-Kinderklinik, Mechernich Kinderklinik, Memmingen Kinderklinik, Minden Kinderklinik, Moers Kinderklinik, Mutterstadt Kinderarztpraxis, Mönchengladbach Elisabeth-Krankenhaus Rheydt, Kinderklinik, München Kinderarztpraxis, München von Haunersche Kinderklinik, München-Gauting Kinderarztzentrum, München-Harlaching Kinderklinik, München-Schwabing Kinderklinik, Münster St. Franziskus Kinderklinik, Münster Uni-Kinderklinik, Münster pädiat. Schwerpunktpraxis, Neuburg Kinderklinik, Neunkirchen Marienhausklinik St. Josef Kohlhof, Kinderklinik, Neuss Lukaskrankenhaus Kinderklinik, Neuwied Marienhaus Klinikums St. Elisabeth, Nürnberg Cnopf‘sche Kinderklinik, Nürnberg Klinik für Neugeborene, Kinder & Jugendliche, Oberhausen Kinderklinik, Oberhausen Kinderarztpraxis, Offenbach/Main Kinderklinik, Offenburg Kinderklinik, Oldenburg Kinderklinik, Oldenburg Schwerpunktpraxis, Osnabrück Kinderklinik, Oy-Mittelberg Hochgebirgsklinik Kinder-Reha, Paderborn St. Vincenz Kinderklinik, Papenburg Marienkrankenhaus Kinderklinik, Passau Kinderarztpraxis, Passau Kinderklinik, Pforzheim Kinderklinik, Rastatt Gemeinschaftspraxis, Ravensburg Kinderklink St. Nikolaus, Regensburg Kinderklinik St. Hedwig, Rendsburg Kinderklinik, Reutlingen Kinderarztpraxis, Reutlingen Klinikum Steinenberg, Rheine Mathiasspital Kinderklinik, Rosenheim Innere Medizin, Rosenheim Kinderklinik, Rosenheim Schwerpunktpraxis, Rostock Uni-Kinderklinik, Rotenburg/Wümme Kinderklinik, Rüsselsheim Kinderklinik, Saalfeld Thüringenklinik Kinderklinik, Saarlouis Kinderklinik, Schweinfurt Kinderklinik, Schwerin Kinderklinik, Siegen Kinderklinik, Singen - Hegauklinik Kinderklinik, Spaichingen Innere Medizin, Stade Kinderklinik, Stuttgart Olgahospital Kinderklinik, Suhl Kinderklinik, Sylt Rehaklinik, Tettnang Innere Medizin, Traunstein Diabetologische Schwerpunktpraxis, Trier Kinderklinik der Borromäerinnen, Trostberg Innere Medizin, Tübingen Uni-Kinderklinik, Ulm Uni-Kinderklinik, Vechta Kinderklinik, Viersen Kinderklinik, Waiblingen Kinderklinik, Waldshut Kinderpraxis, Waldshut-Tiengen Kinderpraxis, Weiden Kinderklinik, Weingarten Kinderarztpraxis, Wiesbaden Horst-Schmidt-Kinderkliniken, Wiesbaden Kinderklinik DKD, Wilhelmshaven Reinhard-Nieter-Kinderklinik, Worms Kinderklinik, Wuppertal Kinderklinik
